# Combined immunohistochemistry of β-catenin, cytokeratin 7, and cytokeratin 20 is useful in discriminating primary lung adenocarcinomas from metastatic colorectal cancer

**DOI:** 10.1186/1471-2407-6-31

**Published:** 2006-02-02

**Authors:** Satoshi Ikeda, Masahiko Fujimori, Satoshi Shibata, Masazumi Okajima, Yasuyo Ishizaki, Takeshi Kurihara, Yoshihiro Miyata, Masahiko Iseki, Yosuke Shimizu, Noriaki Tokumoto, Shinji Ozaki, Toshimasa Asahara

**Affiliations:** 1 Department of Endoscopic Surgery and Surgical Science, Graduate School of Biomedical Science, Hiroshima University, 1-2-3 Kasumi, Minami-ku, Hiroshima, Japan; 2 Department of Surgery, Division of Frontier Medical Science, Programs for Biomedical Research, Graduate School of Biomedical Science, Hiroshima University, 1-2-3 Kasumi, Minami-ku, Hiroshima, Japan

## Abstract

**Background:**

It is important to discriminate between primary and secondary lung cancer. However, often, the discriminating diagnosis of primary lung acinar adenocarcinoma and lung metastasis of colorectal cancer based on morphological and pathological findings is difficult. The purpose of this study was to evaluate the clinical usefulness of immunohistochemistry of β-catenin, cytokeratin (CK) 7, and CK20 for the discriminating diagnosis of lung cancer.

**Methods:**

We performed immunohistochemistry of β-catenin, CK7, and CK20 in 19 lung metastasis of colorectal cancer samples, 10 corresponding primary colorectal cancer samples and 11 primary lung acinar adenocarcinoma samples and compared the levels of accuracy of the discriminating diagnosis by using antibodies against these antigens.

**Results:**

Positive staining of β-catenin was observed in all the lung metastasis of colorectal cancer samples as well as in the primary colorectal cancer samples but in none of the primary lung acinar adenocarcinoma samples. Positive staining of CK7 was observed in 90.9% of the primary lung acinar adenocarcinoma samples and in 5.3% of the lung metastasis of colorectal cancer samples, but in none of the primary colorectal cancer samples. Positive staining of CK20 was observed in all the primary colorectal cancer samples and in 84.2% of the lung metastasis of colorectal cancer samples, but in none of the primary lung acinar adenocarcinoma samples.

**Conclusion:**

Combined immunohistochemistry of β-catenin, CK7, and CK20 is useful for making a discriminating diagnosis between lung metastasis of colorectal cancer and primary lung acinar adenocarcinoma. This method will enable accurate diagnosis of a lung tumor and will be useful for selecting appropriate therapeutic strategies, including chemotherapeutic agents and operation methods.

## Background

After a colorectal cancer operation, metastatic liver cancer is the most frequently observed cancer, followed by metastatic lung cancer. However, it is occasionally difficult to determine on the basis of clinical features whether the lung tumor is a primary lung cancer or a lung metastasis of colorectal cancer; this is because there are cases of lung metastasis at the early stages of colorectal cancer and those that occur more than 5 years after a colorectal cancer operation. Furthermore, the pathological features of primary lung acinar adenocarcinoma are similar to those of differentiated colorectal adenocarcinoma. Hence, the correct diagnosis of a lung tumor is important for therapeutic decision-making, such as the selection of chemotherapeutic agents and operation methods.

β-catenin protein is a component of the Wnt signaling pathway that regulates signal transduction in cell growth, motility, and differentiation [1]. Recent studies have shown that alterations in the Wnt signaling pathway, including mutations in the adenomatous polyposis coli (APC), β-catenin and Axin genes, play important roles in the carcinogenesis of various malignant tumors [2]. Mutations in these genes result in accumulation of the β-catenin protein in the cytoplasm and the accumulated β-catenin translocates into the nucleus [3]. Nuclear β-catenin activates T-cell factor/lymphocyte enhancer binding factor (TCF/LEF) family members and promotes the transcriptions of their target genes such as c-myc and cyclin D1 [4,5]. It is interesting to note that alterations in the Wnt signaling pathway exist in almost all cases of colorectal cancer and result in nuclear accumulation of the β-catenin protein [6-8].

Cytokeratin (CK) is one of the components of the intracytoplasmic cytoskeleton. The CKs constitute a family of more than 30 polypeptides that are classified based on their molecular weights and isoelectric points [9]. It has been reported that CK is distributed in a tissue-specific manner and that immunohistochemistry of certain CKs is useful for the determination of tissues of origin in several types of tumors [10].

This study was undertaken to evaluate the clinical usefulness of an immunohistochemical examination of β-catenin as well as that of CK7 and CK20 for the discriminating diagnosis of primary lung acinar adenocarcinoma and lung metastasis of colorectal cancer.

## Methods

### Tissue samples

Between 1992 and 2002, 269 lung operations for primary lung cancer, 33 for lung metastasis of colorectal cancer, and 725 for primary colorectal cancer were performed in the Second Department of Surgery, Hiroshima University Hospital. The pathological finding in 38 primary lung cancer patients was acinar adenocarcinoma. The tissue samples used in this study were obtained from these patients. We analyzed 19 samples from patients with lung metastasis of colorectal cancer, 10 from patients with primary colorectal cancer, and 11 from patients with primary lung acinar adenocarcinoma; this was because, unfortunately, several samples were lost and not available for use in this study. Each sample was histologically assessed by a pathologist based on the classification criteria followed by WHO [11]. Ten tissue samples of primary colorectal cancer were obtained from the corresponding patients who had undergone operations for lung metastasis of colorectal cancer. We also analyzed a tissue sample of lung metastasis of colorectal cancer obtained by needle biopsy from a surgically resected lung tumor.

### Immunohistochemistry of β-catenin, CK7, and CK20

Each tumor section (4 μm in thickness) was deparaffinized and subjected to antigen retrieval by microwaving in 10 mM of citrate buffer (sodium citrate, pH 6.0) for 30 min. The sections were incubated with anti-β-catenin (Transduction Laboratories, Lexington, KY), anti-CK7 (DAKO Corporation, Carpinteria, CA), and anti-CK20 (DAKO Corporation, Carpinteria, CA) monoclonal antibodies at dilutions of 1:250, 1:100, and 1:100, respectively, for 12 h at 4°C; and they were then stained by the avidin-biotin complex method using a Histofine kit (Nichirei, Tokyo, Japan). The results of β-catenin immunostaining were based on nuclear and cytoplasmic staining and those of CK7 and CK20 were based on the cytoplasmic staining of tumor cells. The β-catenin immunostaining results were evaluated by comparing the staining intensities of tumor cells with those of the adjacent non-tumor cells [12]. Immunostaining was defined as positive when more than 10% of tumor cells were stained by each antibody.

## Results

### Immunohistochemistry of β-catenin, CK7, and CK20

**Figure 1:**
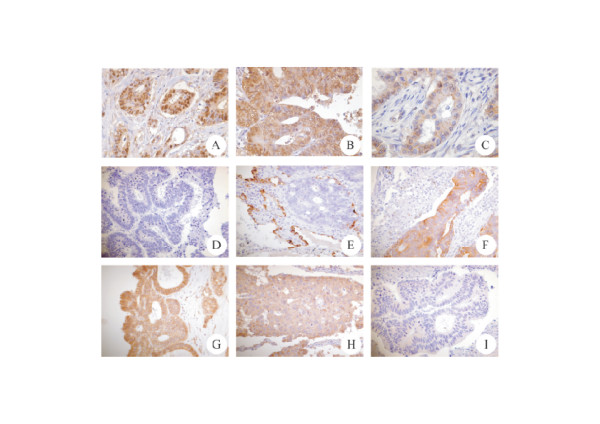
Immunohistochemical analysis of β-catenin, cytokeratin 7, and cytokeratin 20. (A) Positive nuclear staining of β-catenin in primary colorectal cancer. (B) Positive nuclear and cytoplasmic staining of β-catenin in lung metastasis of colorectal cancer. (C) Negative nuclear and cytoplasmic staining of β-catenin and positive membranous staining of β-catenin in primary lung acinar adenocarcinoma. (D and E) Negative staining of cytokeratin 7 in primary colorectal cancer and in lung metastasis of colorectal cancer. (F) Positive cytoplasmic staining of cytokeratin 7 in primary lung acinar adenocarcinoma. (G and H) Positive cytoplasmic staining of cytokeratin 20 in primary colorectal cancer and in lung metastasis of colorectal cancer. (I) Negative staining of cytokeratin 7 in primary lung acinar adenocarcinoma. *Bar *50 μm

β-catenin staining was localized to the cell membrane and was absent from the cytoplasm and nucleus of normal colorectal epithelial cells, normal bronchial epithelial cells, bronchial gland cells, and alveolar cells (data not shown). Positive cytoplasmic and/or nuclear staining of β-catenin was observed in all (100%) the 10 primary colorectal cancer samples as well as in the 19 lung metastasis of colorectal cancer samples, whereas positive nuclear staining was detected in none of the 11 primary lung acinar adenocarcinoma samples (Figure 1A, B, and 1C; Table 1). Three primary lung acinar adenocarcinoma samples showed faint positive staining of β-catenin in the cytoplasm; however, no staining was observed in the nucleus (data not shown). CK7 staining was localized in the cytoplasm in normal alveolar cells, but not in that of normal colorectal epithelial cells. Cytoplasmic staining of CK7 was observed in 10 (90.9%) of the 11 primary lung acinar adenocarcinoma samples but not in 18 (94.7%) of the 19 lung metastasis of colorectal cancer samples nor in any of the primary colorectal cancer samples (Figure 1D, E, and 1F; Table 1). On the other hand, CK20 staining was localized in the cytoplasm in normal colorectal epithelial cells, but not in normal alveolar cells. Positive cytoplasmic staining of CK20 was observed in all (100%) the 10 primary colorectal cancer samples and in 16 (84.2%) of the 19 lung metastasis of colorectal cancer samples, but in none of the primary lung acinar adenocarcinoma samples (Figure 1G, H, and 1I; Table 1).

### Expression patterns of β-catenin, CK7, and CK20

The expression patterns obtained by the immunohistochemical analysis are shown in Table 2. All (100%) the 10 primary colorectal cancer samples and 15 (78.9%) of the 19 lung metastasis of colorectal cancer samples showed a β-catenin + / CK7 - / CK20 + pattern. Of the 19 lung metastasis of colorectal cancer samples, 3 (15.8%) and 1 (5.3%) showed β-catenin + / CK7 - / CK20 - and β-catenin + / CK7 + / CK20 + patterns, respectively. Of the 11 primary lung acinar adenocarcinoma samples, 10 (90.9%) and 1 (9.1%) showed β-catenin - / CK7 + / CK20 - and β-catenin - / CK7 - / CK20 - patterns respectively. We compared the levels of diagnostic accuracy of the stainings in lung metastasis of colorectal cancer samples and in primary lung acinar adenocarcinoma samples. The level of diagnostic accuracy of β-catenin positive staining was higher than that of the other combinations of immunohistochemical patterns. Furthermore, we performed immunohistochemistry on a small sample obtained by needle biopsy, and lung metastasis of colorectal cancer was pathologically diagnosed. The results of immunohistochemistry showed that β-catenin and CK20 were positive and CK7 was negative (data not shown).

## Discussion

It is important to determine whether a lung tumor is primary or secondary lung cancer because the treatment strategies for these two clinical situations are different. However, it is occasionally difficult to make a discriminating diagnosis of primary lung acinar adenocarcinoma and lung metastasis of colorectal cancer in lung tumor patients who have previously undergone colorectal cancer operations. This study was therefore designed to determine whether immunohistochemistry of β-catenin, CK7, and CK20 is useful for the discriminating diagnosis of these two clinical situations.

β-catenin is a key molecule not only in cadherin-dependent cell-cell adhesion but also in the Wnt signaling pathway [13]. The stability of the β-catenin protein is regulated by the Wnt signaling pathway [1]. Glycogen synthase kinase-3β phosphorylates β-catenin, and the phosphorylated form of β-catenin is ubiquitinated and degraded by the proteasome. Thus, the β-catenin protein is maintained at a low level in normal cells. Alterations in the Wnt signaling pathway, including mutations in the APC, β-catenin, and Axin genes, result in an accumulation of β-catenin in the nucleus. The APC gene is defined as a gatekeeper in the adenoma-carcinoma sequence theory of colorectal carcinogenesis because APC alterations, such as mutations and loss of heterozygosity, occur at an early stage of colorectal carcinogenesis [14]. It has been reported that alterations in the APC gene are present in more than 80% of colorectal cancer cases [6,7]. Furthermore, accumulation of β-catenin in the nucleus and/or cytoplasm was detected by immunohistochemistry in 80%–90% of colorectal cancer cases [8,15,16]. Although a small number of the samples were used in the present study, as expected, positive nuclear and/or cytoplasmic staining of β-catenin was observed in all the primary colorectal cancer samples; this is consistent with previous reports. The results of β-catenin immunohistochemistry for lung metastasis of colorectal cancer samples were the same as those for primary colorectal cancer samples. Further, the alterations in the Wnt signaling pathway in lung cancer are unclear. It has been reported that in lung cancer, mutations in the APC gene are not frequent. However, frequent allelic loss at 5q, in which the APC gene is located, and frequent hypermethylation in the promoter region of the APC gene have been reported [17-20]. Although it is not known whether such alterations in the APC gene affect the accumulation of the β-catenin protein in the cytoplasm and/or nucleus, accumulation of β-catenin was observed in none of the primary lung acinar adenocarcinoma samples in the present study. It has also been reported that immunohistochemical positive staining of β-catenin in the nucleus was observed in less than 10% of the primary non-small cell lung adenocarcinoma samples [21], similar to our finding. Our results indicate that immunohistochemistry of β-catenin is useful for the discriminating diagnosis of lung metastasis of colorectal cancer and primary lung acinar adenocarcinoma.

There are many reports on the usefulness of immunohistochemistry of CKs for the discriminating diagnosis of tumors of different origins [22-27]. CKs constitute a family of more than 30 polypeptides and are distributed in a tissue-specific and differentiation-specific manner [9,10]. CK phenotyping by combined immunohistochemistry of several CKs, particularly CK7 and CK20, is commonly used in surgical pathology to determine the origin of a cell type or tissue in which a malignant tumor has developed. It is interesting to note that the CK phenotype is different in each histological type of lung cancer, including adenocarcinoma, squamous cell carcinoma, and small cell carcinoma [28]. It has been reported that CK7 is expressed in 97%–100% of primary lung adenocarcinoma cases and in 5%–27% of primary colon adenocarcinoma cases. In contrast, it has been reported that CK20 is expressed in 7%–10% of primary lung adenocarcinoma cases and 92%–100% of primary colon adenocarcinoma cases [10,23,26]. We also performed immunohistochemistry of CK7 and CK20 in our samples, and our results are consistent with those reported previously. The most common staining pattern of lung metastasis of colorectal cancer is β-catenin + / CK7 - / CK20 +. Of the 19 lung metastasis of colorectal cancer samples used in our study, 78.9% showed β-catenin + / CK7 - / CK20 +. We analyzed several combinations of β-catenin, CK7, and CK20 immunohistochemical patterns. The level of accuracy of the discriminating diagnosis of lung metastasis of colorectal cancer and primary lung acinar adenocarcinoma based on the results of immunohistochemistry of β-catenin alone was higher than that of the discriminating diagnosis based on immunohistochemistry of other combinations. However, it is noteworthy that approximately 10% of primary colorectal cancer and primary lung cancer samples showed negative and positive staining of β-catenin, respectively [8,21]. Furthermore, several samples showed uncommon staining patterns of CK7 or CK20. Taken together, the results suggest that combined immunohistochemistry of β-catenin, CK7, and CK20 is useful for obtaining a more accurate discriminating diagnosis than that obtained by using immunohistochemistry of one of these antigens alone or in other combinations. In addition to β-catenin, CK7, and CK20, thyroid transcriptional factor-1 (TTF-1) may be a useful marker for the discriminating diagnosis of lung metastasis of colorectal cancer and primary lung acinar adenocarcinoma. TTF-1 is a tissue-specific transcriptional factor that plays pivotal roles in the differentiation and morphogenesis of the lung and thyroid. It has been reported that positive staining of TTF-1 was observed in 70%–90% of primary lung cancer cases, but not in lung metastasis of colorectal cancer cases [29-31]. Since CK7 and TTF-1 are specific markers for primary lung cancer and CK20 and β-catenin for colorectal cancer, combined immunohistochemistry of these markers may be valuable in discriminating diagnosis. However, it is important to note that mucinous bronchioloalveolar carcinoma, which is a subtype of lung adenocarcinoma, shows a different CK phenotype. It has been reported that 60% of mucinous bronchioloalveolar carcinoma cases showed CK7 + / CK20 + and that it is difficult to distinguish mucinous bronchioloalveolar carcinoma from mucinous colorectal adenocarcinoma metastatic to the lung based on the CK7 and CK20 phenotype [32]. Furthermore, by using material that was obtained from a tumor of lung metastasis of colorectal cancer by needle biopsy, we succeeded in making an accurate discriminating diagnosis based on the results of immunohistochemistry of β-catenin, CK7, and CK20. Transbronchial lung biopsy and CT-guided biopsy are often performed to obtain a pathological diagnosis of lung tumors. However, the amount of material obtained is occasionally insufficient to enable a discriminating diagnosis of primary and secondary lung cancers based on pathological findings. However, immunohistochemical examinations can be easily performed using only a small amount of material, and the results enable accurate diagnosis in cases of lung tumors.

## Conclusion

Combined immunohistochemistry of β-catenin, CK7, and CK20 is useful for making a discriminating diagnosis of lung metastasis of colorectal cancer and primary lung acinar adenocarcinoma. It is often difficult to distinguish a lung tumor from primary lung acinar adenocarcinoma or lung metastasis of colorectal cancer on the basis of pathological and morphological features, particularly when a small amount of material is obtained. Immunohistochemistry with β-catenin as well as CK7 and CK20 antibodies is helpful for making a discriminating diagnosis, and an accurate diagnosis is important for appropriate selection of therapeutic strategies in cases of lung tumors.

## Competing interests

The author(s) declare that they have no competing interests.

## Authors' contributions

SI, MF, and SS participated in the immunohistochemical analysis. MO and TA drafted the manuscript. YI and SO performed statistical analysis. SI and TK participated in the study design and coordination. YM, MI, YS, and NT selected the tissue samples. All the authors have read and approved the final manuscript.

## Pre-publication history

The pre-publication history for this paper can be accessed here:



## Figures and Tables

**Table 1:** Results of Immunohistochemistry of β-catenin, CK 7 and CK 20.

Samples	Total	β-Catenin (%)	CK 7 (%)	CK 20 (%)
Primary colorectal cancer	10	100	0	100
Lung metastasis from colorectal cancer	19	100	5.3	84.2
Primary lung acinar adenocarcinoma	11	0	90.9	0

CK, cytokeratin

**Table 2:** Expression Patterns of Immunohistochemistry of β-catenin, CK 7 and CK 20.

Pattern of immunohistochemistry					
β-catenin/CK 7/CK 20			Primary colorectal cancer (%)	Lung metastasis from colorectal cancer (%)	Primary lung acinar adenocarcinoma (%)
+	-	+	100	78.9	0
+	-	-	0	15.8	0
+	+	+	0	5.3	0

-	+	-	0	0	90.9
-	-	-	0	0	9.1

CK, cytokeratin
